# Rhabdomyolysis Without Acute Kidney Injury in a 14-Year-Old Child With a Sedentary Lifestyle

**DOI:** 10.7759/cureus.73381

**Published:** 2024-11-10

**Authors:** Muhammad Y Nawaz, Raza Hamdani, Mishal K Siddiqui, Neel Patel, Khushmi Shah, Lori Langdon

**Affiliations:** 1 College of Osteopathic Medicine, Campbell University School of Osteopathic Medicine, Lillington, USA; 2 Pediatrics, Campbell University School of Osteopathic Medicine, Lillington, USA; 3 Pediatrics, Campbell School of Osteopathic Medicine, Lillington, USA

**Keywords:** creatine kinase, kidney injury, liver enzymes, pediatrics, rhabdomyolysis

## Abstract

A 14-year-old African American female patient presented to the emergency department with moderate right calf pain of unknown origin. The pain was present for a couple of days without radiation and progressed with an inability to bear weight. Physical examination revealed tenderness to palpation over the right calf. The patient had no history of overweight, trauma, infection, or extreme physical exertion. She took no medications, supplements, herbals, or used any illegal drugs. Of note, the patient was sedentary. Her blood work revealed elevated creatine kinase and liver enzymes, diagnostic of rhabdomyolysis. All other diagnostic evaluations, including EKG, chest X-ray, leg ultrasound, creatine kinase-MB, urinalysis, thyroid levels, and CBC were unremarkable. No other inherited conditions were identified in lab work. The patient was given dextrose 5% in water with sodium bicarbonate and switched to aggressive hydration via normal saline until discharge.

## Introduction

Rhabdomyolysis is a clinical syndrome characterized by the breakdown of skeletal muscle fibers, resulting in a huge release of creatine kinase and other intracellular factors into the bloodstream. This condition most often results from direct muscle injury but can result from various causes, including trauma, intense physical exertion, drug or toxin exposure, muscle ischemia, neuroleptic malignant syndrome, electrolyte and metabolic abnormalities, genetic anomalies, or heat exhaustion [1]. Any type of uncontrolled rhabdomyolysis can lead to acute kidney injury and other major organ damage. The most common cause in the pediatric population is infection, comprising 30% of cases [2]. The clinical presentation typically includes muscle pain, weakness, and dark urine resulting from myoglobinuria. However, spontaneous rhabdomyolysis, which occurs without a clear precipitating factor, is rare and poses a particular challenge for patient management and treatment [2].

Spontaneous rhabdomyolysis is rare and infrequently documented, though particularly intriguing given the absence of common triggers and known underlying pathophysiology. We present a unique instance of spontaneous rhabdomyolysis in a young patient with no known trauma history or known causative processes. Through a detailed analysis of the clinical presentation and laboratory values, we aim to better understand this uncommon condition and build a stronger differential diagnosis for muscle weakness and pain. We also attempt to underscore the need for further research into the mechanisms of spontaneous rhabdomyolysis [3].

## Case presentation

A 14-year-old female patient presented to the emergency department with worsening right calf pain over three days. Her pain prevented her from ambulating on the right leg, necessitating a limp. She did not have any associated symptoms but notably lives a sedentary lifestyle. She had no history of injury, trauma, or involvement in contact sports. She denied any drug use, prescription or illicit, heat exhaustion, or inherited causes of rhabdomyolysis. She admitted to sitting with her right leg over her left knee for hours while playing video games, the exact location where her pain is localized. She had a history of mild intermittent asthma and allergic rhinitis, which are well-controlled.

Her vital signs included a blood pressure of 120/78 mmHg, heart rate of 100 bpm, temperature of 98.1 °F, BMI of 21.1 kg/m^2^, respiration of 19, and oxygen saturation of 96%. The complete metabolic panel displayed an initial creatine kinase of 7840 with concurrent elevated aspartate aminotransferase (AST) and alanine aminotransferase (ALT) at 106 and 45, respectively (Table 1). The remainder of her diagnostic evaluations, including EKG, chest X-ray, leg ultrasound, creatine kinase-MB, urinalysis, and complete blood count were benign. She was given dextrose 5% in water and switched to normal saline two times for maintenance. She received a 1-liter bolus over an hour, followed by 200 ml/hr overnight. On hospital day two, her creatine kinase went down to 4047, and her ALT normalized with AST down to 55. Her blood urea nitrogen/creatinine ratio was decreased to 10.9 On hospital day three, her right leg calf pain had improved. She was aggressively hydrated and on day eight was ready for discharge with a creatine kinase of 356. She was advised to follow up closely with her primary care physician.

**Table 1:** Case chronology AST: aspartate aminotransferase; ALT: alanine aminotransferase; UA: urinalysis; CK: creatine kinase; CMP: comprehensive metabolic panel

Day -3	Leg/calf pain
Day -2	Worsening leg/calf pain
Day 0	Calf pain is improved. Labs ordered: CBC, CMP imaging ordered: EKG, CXR, Leg X-ray, US, UA, initial CK level 7840. Treatments started: normal saline 2x maintenance. 1-liter bolus followed with 200 ml/hr overnight
Day 1	Calf pain is improved. CK is down from 7294 to 4047. ALT normalized, AST 55
Day 2	CK: 2668.
Day 3	Persistent right calf pain, inability to bear weight
Day 4	CK 545. Right calf improvement, able to move more freely
Day 5	CK 356, discharge

## Discussion

This case demonstrates the efficient and accurate diagnosis of spontaneous rhabdomyolysis in a pediatric patient leading to an ideal patient result. Rhabdomyolysis pathogenesis typically involves trauma to an affected extremity. However, this case's peculiar spontaneous development requires additional investigation into the hereditary and congenital causes of the disease. The following list represents rare genetic disorders that commonly afflict pediatric patients and result in rhabdomyolysis: McArdle disease, aldolase A deficiency; disorders of intramuscular calcium release; and ryanodine receptor 1 deficiency (Table 2). McArdle disease is one of the rare hereditary disorders associated with rhabdomyolysis. This glycogen storage disease typically presents with fever, fatigue, and muscle cramps following intensive physical activity [4]. Similar to the case above, four pediatric patients were diagnosed with rhabdomyolysis after developing increased creatine kinase and myalgias [5]. Despite a similar physical presentation, the four pediatric patients had classic McArdle symptoms that presented after physical activity [6]. In all four pediatric patients from the study above the glycogen phosphorylase, a muscle-associated gene had a distinct homozygous mutation predisposing the population to rhabdomyolysis development. This healthy 14-year-old female should get tested for glycogen phosphorylase, a muscle-associated gene homozygous mutation to rule out a mosaic McArdle diagnosis. These tests were not conducted in the in-patient setting due to a lack of resources.

**Table 2:** Etiologies of rhabdomyolysis

Category		Estimated Percentage	References
Trauma or Muscle Compression	Crush Injuries	8-30%	[1-8]
	Prolonged Immobilization	-	[1,2]
	Compression of Blood Vessels		
	Surgery	4-12%	[1,2,9-12]
	Compartment Syndrome	10-20%	[1,2,13]
	Electrical Injuries and Burns	1-5%	[1,2,14-15]
Nontraumatic-Exertional	Physical Exertion	10-30%	[1,2,16-21]
	Impaired Heat Loss	-	[1,2]
	Hypokalemia		
	Sickle Cell Trait		
Nontraumatic-Nonexertional	Drugs and Toxins	10-15%	[1,2,22-28]
	Infections	2-10%	[1,2,23,29-32]
	Electrolyte Abnormalities	-	[1,2]
	Endocrine Disorders		
	Inflammatory Myopathies		
Inherited Conditions	Metabolic Myopathies	-	[1,2]
	Muscular Dystrophies and Congenital Myopathies		
	RYR1 Pathogenic Variants		

Our patient’s profound creatine kinase elevation indicates a more acute disease development process. According to the patient's history, she had not experienced similar symptom progression. The findings from this study are identical to that of another previously published case concerning a pediatric 14-year-old male with aldolase A deficiency [7]. The case explores another rare glycogen storage disease that resulted in multiple rhabdomyolysis emergencies and subsequent hemolytic anemia in a pediatric patient. Although the aldolase A deficient patient also had nontypical seizure development, both patients' laboratory evaluations revealed elevated AST, ALT, and creatine kinase. HyperCKemia and elevated liver enzymes in both cases raise suspicion for similar etiologies and treatment protocols. In contrast to the 14-year-old female who was treated with normal saline and rest, the aldolase A-deficient 14-year-old male was treated with a ketogenic diet and anti-seizure medications. Further investigation into the genes encoded by aldolase isozymes in the 14-year-old female can elucidate an explanation for the acute rhabdomyolysis seen in the pediatric patient.

An important finding from our 14-year-old patient is the lack of acute kidney injury even with acute hyperCKemia. A variety of triggers can precipitate increased CK, myalgias, and rhabdomyolysis but often result in acute kidney injury. Disorders of intramuscular Ca2+ release - ryanodine receptor 1 and calcium voltage-gated channel subunit alpha 1S dysfunction - are two of the most common causes of the symptoms seen in this case [8,9]. Although these two intramuscular Ca2+ disorders often present with kidney injury, hyperkalemia, and unprompted rhabdomyolysis require additional genetic testing to exclude a disorder that can result in acute renal failure. Severity can range from minor increases in serum muscle enzymes to critical conditions involving extreme enzyme elevations, electrolyte imbalances, and acute kidney injury [10].

The classic triad of rhabdomyolysis includes muscle pain, weakness, and dark urine, though this complete triad is only observed in a minority of cases [11-13]. Muscle pain and weakness often develop over hours to days and predominantly affect proximal muscle groups such as the thighs, shoulders, and lower back. Muscle swelling may also occur, particularly following fluid rehydration [14,15-17]. Dark urine, ranging from red to brown or "tea-colored," is a key indicator but is present in fewer than 10% of patients. Urinalysis is crucial to differentiate myoglobinuria from hematuria [18,19].

Rhabdomyolysis can lead to severe complications, including fluid and electrolyte disturbances such as hypovolemia, hyperkalemia, hyperphosphatemia, and hypocalcemia. These imbalances result from the release of muscle cell contents and may lead to acute kidney injury, affecting a significant proportion of patients [20-23]. Other potential complications include compartment syndrome, which can exacerbate muscle damage due to increased pressure within muscle compartments, and in rare instances, disseminated intravascular coagulation [13,24-25]. Additionally, patients may experience liver dysfunction, altered mental status due to electrolyte disturbances or underlying causes, and respiratory issues [26-30]. Severe electrolyte imbalances can also lead to cardiac dysrhythmias and an increased risk of cardiac arrest [31].

Rhabdomyolysis should be considered in patients exhibiting muscle pain, weakness, and dark-colored urine, although the full triad of symptoms is infrequently observed [10]. Diagnostic evaluation is recommended for individuals presenting with muscle pain and discolored urine. Suspected rhabdomyolysis is also warranted in patients with potential risk factors or triggering events, even if muscle pain or pigmented urine is not present, as symptoms may be subtle or absent in up to 50% of cases. This includes scenarios involving unconsciousness or altered mental status [32].

The clinical history should assess potential causes and risk factors for rhabdomyolysis, including recent trauma, use of myotoxic medications, substance abuse, prolonged immobilization, recent surgery, infections, strenuous exercise, heat exposure, pre-existing muscle disorders, and endocrine diseases [33-36]. Physical examination should focus on muscle weakness, tenderness, swelling, redness, and signs of trauma or compartment syndrome.

Essential diagnostic tests done in our case were EKG, chest X-ray, leg ultrasound, creatine kinase-MB, urinalysis, and complete blood count. Additional tests may include a complete blood count, renal function tests, electrolytes, liver enzymes, coagulation studies, arterial blood gas, urine dipstick to detect myoglobinuria, and blood cultures if an infection is suspected. Toxicology screens may be required if substance abuse is a concern [32]. A different published case discussing rhabdomyolysis in a 38-year-old male patient with COVID-19 utilized APT, activated partial thromboplastin, lactate dehydrogenase, and C-reactive protein in the care period for the patient [30]. Lactate dehydrogenase and C-reactive protein may help quantify the extent of muscle damage or inflammation, but they’re not specific. An activated partial thromboplastin is useful in the ICU setting where bleeding risk may be critical but is not a standard test used in rhabdomyolysis [16].

Elevated creatinine kinase levels are indicative of rhabdomyolysis, with values typically ranging from 1500 to over 100,000 units/L, and often exceeding five times the normal upper limit. Creatinine levels rise within 2 to 12 hours after muscle injury, peak within 24 to 72 hours, and then decline over a few days [37,38]. Myoglobinuria, while characteristic, may be absent due to its rapid clearance compared to creatinine kinase. The presence of myoglobin in urine is confirmed by a positive dipstick test for heme with minimal red blood cells on microscopic examination [11,39].

In addition to elevated Creatinine kinase, other muscle enzymes such as aldolase and lactate dehydrogenase may be elevated, though they do not specifically diagnose rhabdomyolysis but indicate muscle injury. Serum aminotransferases are often elevated in rhabdomyolysis but can also suggest liver damage. Diagnosis is confirmed by the presence of characteristic symptoms, risk factors, elevated creatinine levels, and evidence of myoglobinuria [40,41].

## Conclusions

Spontaneous rhabdomyolysis should be considered as a potential differential when a patient presents with elevated creatine kinase and liver function tests over a short period of time with no history of acute trauma or genetic factors. Early detection and treatment with aggressive hydration and pain management can help reduce the duration of disease progression. The findings from this case reveal a potential link between prolonged sedentary behavior with crossed legs and the atypical development of spontaneous rhabdomyolysis. The findings from this case should prompt further investigation into the pathophysiology of this disease.
